# Sensitizing Black Adult and Youth Consumers to Targeted Food Marketing Tactics in Their Environments

**DOI:** 10.3390/ijerph14111316

**Published:** 2017-10-29

**Authors:** Katherine Isselmann DiSantis, Shiriki Kumanyika, Lori Carter-Edwards, Deborah Rohm Young, Sonya A. Grier, Vikki Lassiter

**Affiliations:** 1 Department of Public Health, Arcadia University, 450 S. Easton Rd, Glenside, PA 19038, USA; disantisk@arcadia.edu; 2 Department of Community Health and Prevention, Drexel University Dornsife School of Public Health, 3215 Market Street, Philadelphia, PA 19104, USA; 3 Public Health Leadership Program, Gillings School of Global Public Health, University of North Carolina, 4111 McGavran-Greenberg Hall, CB #7469, Chapel Hill, NC 27599, USA; lori_carter-edwards@unc.edu; 4 Department of Research and Evaluation, Kaiser Permanente Southern California, 100 S. Los Robles, 2nd Floor, Pasadena, CA 91101, USA; Deborah.R.Young@kp.org; 5 Kogod School of Business, American University, 4400 Massachusetts Ave, NW Washington, DC 20016, USA; griers@american.edu; 6 Social Science Research Council, One Pierrepont Plaza (300 Cadman Plaza West), 15th floor, Brooklyn, NY 11201, USA; lassiter@ssrc.org

**Keywords:** targeted food marketing, obesity, Black American health, health disparities, consumer perceptions, food policy, food environment

## Abstract

Food marketing environments of Black American consumers are heavily affected by ethnically-targeted marketing of sugar sweetened beverages, fast foods, and other products that may contribute to caloric overconsumption. This qualitative study assessed Black consumers’ responses to targeted marketing. Black adults (2 mixed gender groups; total n = 30) and youth (2 gender specific groups; total n = 35) from two U.S. communities participated before and after a sensitization procedure—a critical practice used to understand social justice concerns. Pre-sensitization focus groups elicited responses to scenarios about various targeted marketing tactics. Participants were then given an informational booklet about targeted marketing to Black Americans, and all returned for the second (post-sensitization) focus group one week later. Conventional qualitative content analysis of transcripts identified several salient themes: seeing the marketer’s perspective (“it’s about demand”; “consumers choose”), respect for community (“marketers are setting us up for failure”; “making wrong assumptions”), and food environments as a social justice issue (“no one is watching the door”; “I didn’t realize”). Effects of sensitization were reflected in participants’ stated reactions to the information in the booklet, and also in the relative occurrence of marketer-oriented themes and social justice-oriented themes, respectively, less and more after sensitization.

## 1. Introduction

The most recent dietary recommendations report from public health experts in the United States focused on influences arising from food environments, including marketing influences, such as promotion, pricing, and placement of products [1]. This is an echo of past calls to develop a comprehensive strategy to address disparities in obesity, particularly childhood obesity, and other diet-related diseases [2,3]. Recent data illustrate that racial disparities in obesity persist, despite numerous policy initiatives aimed at reducing obesity across the population. Black children and adolescents have a prevalence of obesity of 20% versus 15% in White children and adolescents [4]. Black adults also have a higher prevalence of obesity relative to Whites (48% vs. 36%) [5]. Indicative of disparities in obesity-related chronic diseases, Black Americans aged 35 to 64 are 50% more likely to have high blood pressure and 65% more likely to have diabetes, compared to Whites of the same age [6].

Food marketing environments of Black American consumers are heavily affected by ethnically-targeted marketing of sugar sweetened beverages, fast foods and other products that may contribute to caloric overconsumption [7,8]. Marketing of such products is a characteristic of food environments in general [9]. Exposure to such marketing is above average in the physical, media, and social environments of Black Americans and is tailored to resonate with and leverage Black cultural preferences [10]. These marketing patterns may promote caloric overconsumption and likely influence eating patterns and obesity-related health risks [8]. Countering these marketing practices is, therefore, of high interest from the perspective of addressing obesity and related health disparities that affect Black communities.

How to counter targeted marketing of unhealthy foods to Black communities or other ethnic minority populations is far from straightforward [11]. Effective counter marketing in the food arena is extremely challenging in general [12,13]. Food is indispensable, and it has been difficult to classify any particular food or food category as toxic, in the sense that applies to a product like tobacco. Moreover, food is big business on a grand scale; marketing of high-calorie food products is pervasive and driven by a strong profit motive; and food advertising and promotion are protected by law under the First Amendment [14]. Targeting and tailoring products to fit the preferences and needs of specific consumer “segments” is standard marketing practice, and includes the use of ethnicity as a basis for targeting. Public health arguments are made for restricting marketing of high-calorie foods and beverages to children on the basis of their age or developmental stage, but arguments about restricting marketing on the basis of ethnicity falter on the premise that people in ethnic minority populations are somehow less able to fend for themselves in the marketplace [11]. Thus, at least at present, the main recourse, from a public health perspective, is to point out issues related to targeted marketing with the expectation of mobilizing those affected to respond with resistance and advocacy for a change in business practices.

A study conducted by the Rudd Center indicated that this approach might be effective when parents are made aware of targeted marketing to their children [15]. However, evidence from consumer research indicates that marketing, including ethnically targeted marketing, is not always viewed as problematic, and also may not be seen by consumers as particularly influential on their behavior [10,16]. In fact, for ethnic groups or other populations that have been historically excluded from, or less valued in the mainstream marketplace, views of ethnic marketing may be positive—signaling recognition and respect. It is within this context that we undertook a study to explore responses to targeted marketing among Black consumers, in an effort to inform appropriate and potentially effective counter marketing strategies. This study sought to understand the views of Black American consumers on target marketing, including its relevance as a social justice issue. Given that limited research has examined Black perceptions of targeted food marketing, and based on results of a pilot study conducted in Philadelphia, we designed a qualitative study which incorporated sensitization procedures within focus groups. Sensitization procedures are used as a critical practice to understand social justice concerns [17]. In the current study, sensitization took place after an initial focus group. Participants then returned after sensitization to allow an understanding of whether and how their views evolved.

## 2. Materials and Methods

### 2.1. Participant Recruitment

Black adults and youth were recruited in Durham, North Carolina, and Prince George’s (PG) County, Maryland, in partnership with a local community-based organization at each location. These areas were chosen because of their involvement in a larger multi-site research project of the African American Collaborative Obesity Research Network (AACORN), and to take advantage of infrastructure developed during prior collaborations. The multi-site nature of the project was intended to allow for more demographic diversity among participants than would have occurred if focus groups were only conducted in one area. PG and Durham Counties are located in the mid-Atlantic and Southeastern United States, respectively, and have different sociodemographic profiles [18]. Durham (~300,000) is about one-third of the size of PG County (~900,000) in terms of population size, with about half the population density per square mile compared to PG County. Compared to Durham, PG county has a higher percentage of Black Americans (63% vs. 37%), fewer people living in poverty (10% vs. 19%), similar adult obesity prevalence (33% vs. 28%), and similar proportions of low-income residents with low access to healthy foods (4% vs. 6%).

Staff at the collaborating community centers, where the focus groups were to be held, advertised the focus groups to their clients, and in the surrounding communities through flyers and word of mouth. Eligible participants were invited to discuss food and beverage marketing in their communities, and were told participation required attendance at one 60–75 min and one 30–45 min focus group, separated by about one week. Youth (ages 16–18) and adults (ages 19 and older) were recruited for separate groups, to allow for the possibility that youth and adults might have different marketing exposures, view food marketing issues differently, or respond differently in the presence of adults. Descriptive data were collected with anonymous questionnaires.

### 2.2. Study Design 

Each site conducted four sets of focus groups (two with mixed gender groups of adults, and two with gender-specific groups of youth). Focus groups are designed to elicit responses and thoughts on a topic, while allowing for deeper discussion and interaction among respondents [19], which fit well with the purpose of this project. Each group met twice. The first focus groups elicited participant responses to scenarios about various targeting marketing tactics (pre-sensitization). Participants were then asked to review a provided informational booklet about targeted marketing to Black Americans (sensitization) before returning for the focus group one week later (post-sensitization). Focus groups were moderated by a professional from the local community; both moderators were Black Americans. All groups used the same procedures and moderator guide, except that some scenarios differed for youth. Discussions were recorded and professionally transcribed. The study procedures were approved by the institutional review boards of the University of Pennsylvania (University of Pennsylvania Institutional Review Board, Social and Behavioral Sciences, Protocol #810296; Approved 8-3-2010); Duke University (Duke University Medical Center Institutional Review Board, IRB #11-2068; approved in 2011), and the University of Maryland Institutional Review Board (Application #10-005, approved 03/02/2010).

### 2.3. Data Collection Procedures 

#### 2.3.1. Pre-Sensitization Focus Group Protocol

**Figure 1:**
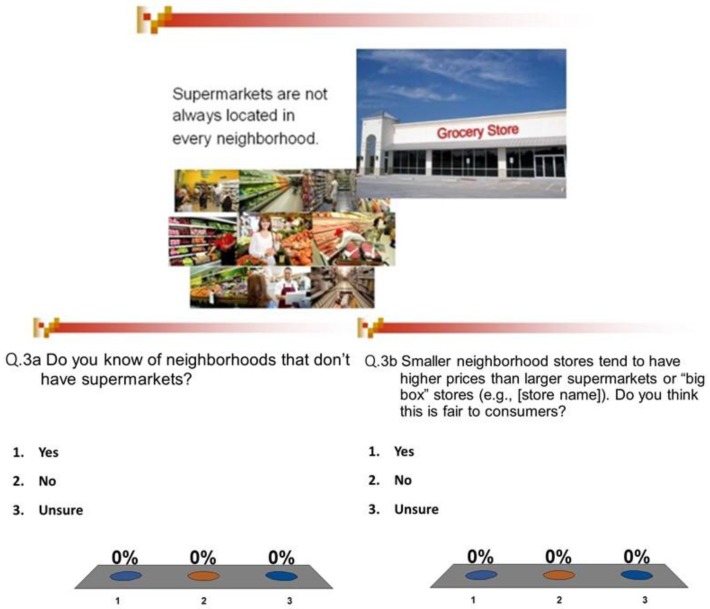
Example image of moderator guide slides that participants saw and responded to initially via clicker technology, allowing display of anonymous response counts to stimulate discussions on specific food and beverage marketing approaches.

During the “pre-sensitization” focus group, participants were presented with scenarios focused on examples of marketing tactics that target Black Americans with high-calorie, low-nutrition foods through pricing, place (availability), products, and promotion (i.e., the “4 Ps” of marketing). The general technique of posing scenarios that allow presentation and manipulation of various issues is common in marketing research. The concept for the scenarios was based on a study by Smith and Cooper-Martin [20], in which these authors explored ethical concerns about consumer vulnerability and product harmfulness in relation to target marketing strategies. The scenarios developed for this study were sometimes factual, and other times contrived, but realistic situations drawn from research and observed marketing practice. Scenarios were projected as Microsoft PowerPoint slides and the moderator read the information on the slide aloud. A typical slide would have 1–2 images, and a small amount of text describing the tactic. An example can be seen in Figure 1. Table 1 lists all of the scenarios used, and the moderator’s questions posed after scenarios were presented. As indicated, some scenarios presented policy solutions to combat marketing tactics thought to contribute to obesity. The moderator used “polling” or “personal response system” software technology (Turning Point Technologies, LLC, Youngstown, OH, USA) that allowed participants to use hand-held clickers to respond initially to questions posed with their reaction to the marketing tactic being presented; data were then immediately displayed anonymously as a bar graph of the distributions of responses. Then, broader explanations and discussion of reactions were sought through group dialogue. Poll responses were not tallied or retained; the purpose was to stimulate thinking and discussion.

#### 2.3.2. Sensitization Procedure

Results of pilot focus groups with adults and youth in Philadelphia indicated that a “consciousness raising” approach could be informative [16]. In the present study, the sensitization was a 12-page booklet (see Supplementary Materials). Participants were given the booklet at the end of the first focus group, and asked to take it home and review and share with others as they wished. The booklet described the intent of food and beverage marketing tactics, organized according to the four marketing Ps (product, price, placement, promotion), and focused on four of the scenarios presented in the pre-sensitization focus group (Table 1). In part, this was designed to improve marketing literacy, but the sensitization to ethnic targeting also potentially raised issues of fairness.

#### 2.3.3. Post-Sensitization Focus Group Protocol

The post-sensitization focus group occurred about one week after receiving the booklet. The goal was to understand participants’ reactions to the information in the booklet, re-assess their perceptions about food and beverage marketing tactics discussed in the initial focus group, and determine whether sensitization had changed their perceptions relative to their initial reactions. Polling technology was not used during this less structured, follow-up focus group. Moderators first reminded participants of the information presented in the sensitization booklet, and then distributed slides (printed one slide per page) that presented the food and beverage marketing scenarios discussed in the pre-sensitization group. Participants were asked to review the scenarios and those in the booklet (copies were provided during the group), and were asked, “Is there any scenario there that stood out in your mind?” Additional questions were designed to allow participants to describe how their reactions to the scenarios might have evolved since the pre-sensitization focus group, for example: “Has your opinion about any of the scenarios we showed previously changed after reading the booklet?”, and “What did you learn from the booklet that informs your opinion now?”. The final question of post-sensitization focus groups aimed to understand what food and beverage marketing participants would want in their community: “In relation to foods and beverages what types of products would you like to see advertised in your communities?”.

### 2.4. Data Analysis

Written transcripts for each site were reviewed by the respective field site investigators and their team, as well as by researchers at the AACORN national office. Conventional content analysis was used to identify views of participants overall, including those that reflected or disagreed with perceptions that targeted marketing practices were unfair, and constituted a social justice issue. Conventional content analysis involves “open-coding” of text transcribed from focus groups to identify segments of text that reflect convergent and divergent ideas within and across transcripts. These codes then led to themes, which emerge from the data in an inductive manner [21]. Two team members, who did not moderate the focus groups, performed the coding. Site-specific codes were developed across pre- and post-sensitization focus groups, and an overall codebook (a dictionary with a brief definition of each label used) was developed. Checks of intercoder consistency were conducted when a minimum of 30% of transcripts had been coded for each site. Codes used differently by different coders were reviewed and revised to align those already coded and to achieve clarity for the remainder of the coding process.

The NVivo software (QSR International Pty Ltd, Richmond, UK) was used to facilitate the process of grouping data into categories and subcategories. During this phase, thematic development was assessed for salience in pre- and/or post-sensitization groups. The codebook and emerging themes were reviewed and discussed with the respective field site investigative teams and the AACORN Research, Evaluation, and Dissemination Core research team. These discussions led to both confirmation and further refinement of the final codebook and themes. The initial qualitative impressions of changes post-sensitization were further explored by tabulating the use of selected codes pre- and post-sensitization.

## 3. Results

### 3.1. Sample 

Durham participants included 15 adults and 13 youth (7 males and 6 females), and PG County included 15 adults and 22 youth (12 males and 10 females). All youth participants were aged 16–18 years. Table 2 shows demographics for adult participants by site. Adult participants in PG County were older than those in Durham. Most were female and were the primary food shoppers for their households. Compared to those in Durham, more adults in Maryland had college degrees or higher, and more were full or part-time employed. The follow-up rate for the post-sensitization focus groups was 100%.

### 3.2. Themes

Six themes were identified, and are described in detail below with illustrative quotes. We organized themes broadly as related to: seeing the marketer’s perspective (“it’s about demand”; “consumers choose”), respect for community (“marketers are setting us up for failure”; “making wrong assumptions”), and food environments as a social justice issue (“no one is watching the door”; “I didn’t realize”). Results revealed much similarity in youth and adult perspectives; themes identified were not age group-specific. Themes which were solely present in either the pre- or post-sensitization group were allowed, but they did not emerge from the data. Thus, any differences identified between pre- and post-sensitization findings are described within theme descriptions. For context, citations of quotes from transcripts are identified as from adult or youth focus groups, and also by gender for youth. Gender of speakers in the mixed-gender adult focus groups could not be identified from transcripts.

#### 3.2.1. Seeing the Marketer’s Perspective 

##### It’s about Demand

During pre-sensitization groups, many participants pointed to the issue of consumer demand as an explanation for greater availability of unhealthy foods in certain communities. These participants often asserted that some business practices described in the presented scenarios were “good business practices”, and were mainly responses to consumer demand. One youth participant (female) raised this point, **“they (food companies) are not going to target people who do not want it (food product)”,** and also, downplayed worries about high availability of unhealthy items and/or high-priced items in certain communities by saying, **“if they don’t like it, they could drive somewhere else.”** In regard to pricing, participants stated that businesses charge the highest amount they think they can get, and that is a good business practice and not an issue of “fairness”. Many also commented on the principles of supply and demand, pointing out that some scenarios presented, such as high food prices in small neighborhood stores and lack of supermarkets in some neighborhoods, were of less political or social justice relevance and more related to open markets, which cause food prices to be high when availability is low.

Some participants also discussed positive aspects of marketing that targeted a racial/ethnic group. This perspective was found in both adults and youth, although was more pronounced in youth. These participants said that this might result in a product more in line with cultural values and preferences; therefore, targeted marketing could result in more satisfaction with the product. They also described how culture draws you to certain products, with one youth (male) stating, **“It’s not a Black thing”** when describing how different groups might have affinity to different products.

In the post-sensitization focus groups, some sentiments of demand side issues being in the consumer’s control were still asserted, with one adult participant, stating that **“it’s an individual thing before it’s a community thing”** and another adult following up with, **“some people just don’t care. Some people are willing to die for that last pork chop”.** But in most cases, the context around these sentiments seemed to change, where participants expressed more tension between “good business strategies” and the potential impact on the community. For example, one youth participant (male) said in the post-sensitization group, **“it’s a good (business) strategy but at the same time it’s not good for our society”.**

##### Consumers Choose

Some participants emphasized the individual choices which often lead to negative, food-related health issues. These participants expressed the power of individual choice, stating that interventions such as sugar sweetened beverage (SSB) taxation would not change behaviors because, **“If you want a soda, you want a soda”** (adult participant during pre-sensitization). Others even described unintended consequences of SSB taxes, with one participant describing that those taxes on SSBs or “junk food” would result in people spending a greater percentage of their food budget on those items, leaving them less money to spend on healthy foods. There were also some references to community members being “addicted” to certain “unhealthy foods” such as fast foods and SSBs. They also described some motives of consumers, including wanting quick and convenient options that are either “on-the-go” or do not require cooking. Desires like these were connected to less healthy food purchases. For example, one adult participant said, **“I think people going to still go fast foods sometimes because you just don’t want to cook it”.**

During the post-sensitization, focus on consumer responsibility was less pervasive. Participants began to mention environmental challenges faced by consumers, such as lower pricing of unhealthy items, time pressures on families, and low availability/promotion of healthier options, to acknowledge the difficulty of making the “healthy” choice. This statement by a youth participant (female) illustrates this tension between individual responsibility and the environmental features of a community that promotes unhealthy choices: **“But if you don’t have a supermarket in your neighborhood, then you’re more likely to go to those fast food places because it’s quick. And then a lot of times parents, they work late. So it’s—it’s really easy to just stop by somewhere, pick up a whole meal, come home, feed the kids, and do everything that you’ve got to do even though we should all eat more fresh fruits and vegetables”.** And some reiterated that businesses are focused on profits, and that individuals need to recognize their “choice”: **“but you can’t stop a person from making a living, that’s what they’re doing, this is your choice to go there”** (adult participant in post-sensitization group)**.** Participants talked more positively about taxation approaches particularly when discussing how the money could be used to “fight back” against marketing and to help stop the obesity crisis.

#### 3.2.2. Respect for the Community

##### Marketers are Setting us up for Failure

While adult and youth participants appreciated the profit-driven motives and business strategy of targeted marketing, many expressed the perception that targeting a racial/ethnic group was **“wrong”**. One youth participant (female) acknowledged this as a business strategy, while questioning the justice of the approach during the post-sensitization group: **“It’s smart, but wrong”.** As scenarios portrayed approaches of targeted marketing, participants would comment that the scenario got them **“thinking”.** This was particularly observed in the post-sensitization focus groups where participants expressed they had been taking notice of marketing tactics in their environment in the time since the pre-sensitization group. They expressed frustration with some marketing tactics during both the pre- and post-sensitization groups, saying: **“They are setting us up for failure”**, **“They do not look at us as actual human beings, just as a profit”** and **“why it got to be a Black neighborhood so it makes me angry but… I know we still got a choice”.**

Two scenarios were met with substantial criticism when first introduced in the pre-sensitization focus group: celebrity endorsement of products and product development targeted at certain racial/ethnic groups. On celebrities in advertisements, some participants stated these were often **“false advertisements”**, as it portrayed slim celebrities promoting calorie dense foods. And one adult wondered during the post-sensitization group whether marketers could use celebrities to promote healthier options: **“if they’re going to advertise using these celebrities, advertise them eating a carrot”.** Regarding specialized products, many participants felt like a product targeted to a racial/ethnic group was equivalent to **“altering”** a product, and this was met with negative views regarding the food/beverage company. One adult participant stated, while reflecting on this issue, **“if they are going to do that to our drinks… how far will they go?”**. And others expressed a need to know what was in a product, and to not mislead consumers on the ingredients or nutrient make-up of a food or beverage. This led some participants to discuss a need for more government oversight in the marketing of products in order to have consumers understand what exactly is in the product they are purchasing.

Some adult participants connected targeted marketing to a broader social justice issue during both the pre- and post-sensitization groups. Numerous adults stated that targeted marketing is an example of outside entities **“trying to kill us”** (“us” is referring to the Black community). They commented that food marketing approaches highlighted in the focus group and in the sensitization booklet might result in worse health and early death, citing health conditions such as hypertension and diabetes.

##### Marketers’ Assumptions are Wrong

Participants discussed thinking that marketers are making false assumptions about preferences of Black consumers, which was observed during both the pre- and post-sensitization groups. They felt that Black consumers are assumed not to want healthier or fresher options. In these conversations, participants also expressed being insulted by the assumptions behind the marketing strategies presented. One youth participant (male) expressed his concerns, **“It matters to me because I don’t want to be stereotyped because like okay he is Black so he is going to go to Church’s** (referring to Church’s Chicken, a quick serve restaurant chain) **every Friday for fish specials”.** Regarding assumptions about whether Black consumers want healthier options, some participants noted that individual choice is involved, but that it seemed marketers are focused on making less healthy options enticing for Black consumers.

Adult participants expressed concern about strategies that target youth, as they felt youth were being manipulated. One adult described the tactic: **“going for that younger generation with using a young celebrity to bring in to make it popular with the younger kids”.** And another adult said, when describing a popular professional athlete endorsing a flavored sugar-sweetened sports water, **“because you can hear, “Mommy, I want this because (Athlete’s name) had it and I want to be a basketball player just like him” so I think marketing has a huge effect”.**

Participants also raised concerns that certain marketing strategies might target particularly vulnerable sub-populations of the Black community. An example given was “value” menu platforms, which offer select items at low prices (e.g., $1.00). An adult participant described how such pricing strategies “prey” on the circumstance of lower income and/or single-parent families, where low cost, quick and easy options are needed, but that these options are all unhealthy. When describing the placement of fast food restaurants and their use of value priced menus, she said it is like **“a trap for our people”**. Many participants also raised the importance of food store availability in response to scenarios depicting neighborhoods without supermarkets. Particular concern was raised for community members who did not have cars or other means of efficient transportation, stating that this was **“unfair”** or making it hard for these individuals to access healthier options.

#### 3.2.3. Food Environments as a Social Justice Issue

##### No One is Watching the Door

This theme ran contrary to the theme “Consumers Choose”, and was most pervasive in the post-sensitization group. Some participants connected the marketing of unhealthy foods to larger social justice issues. One adult participant spoke in frustration during the post-sensitization group about the marketing tactics, saying **“nobody’s watching the door, in terms of what’s being put out into the community”**, and that unhealthy options were prevalent in the community. In these cases, participants spoke with some frustration that government officials and/or other leaders were not **“minding”** (in the sense of monitoring) the actions of businesses with the intent to protect community health. Other non-food-related threats to the Black community were also discussed in this context, including a lack of response or slowed responses by officials to problems in Black communities relative to non-Black communities. Others pointed out the need for community members and individual consumers to take a stand against the marketing of unhealthy foods, particularly during the post-sensitization groups. One adult stated that people need to speak up because individuals have more influence than they currently utilize. A youth participant (male) spoke with frustration, saying **“Don’t wait until there is a problem then you can’t solve it. Have half of the world population be dead by the time you cure it”.** And another youth participant (male) pointed out that it is difficult to activate community members: **“I know some activities and stuff you involve yourself in that make you more susceptible to it but like he said until people actually—until it actually affects them personally I think for the most part a large part of the population won’t actually take it seriously”.** Others raised concerns about government involvement in obesity prevention efforts, citing either lack of oversight or suggesting ways to alter current government strategies to make them more effective. For example, one youth participant (female) said during the post-sensitization group: **“Why wouldn’t they just lower the taxes and stuff for that healthy food”.** Another adult reflected during the post-sensitization group, “**it’s bringing to my attention is just how shabbily the U.S. government regulation people are, in terms of what they allow people to feed us”.** Participants suggested numerous approaches where the emphasis would be on increasing promotion of and availability of healthier options, while reducing prices of these foods.

##### I Didn’t Realize

In post-sensitization sessions, many participants found the booklet informative because it had details on food marketing strategies. Participants said they felt the booklet provided them data (often referring to “statistics”) which made them more aware of marketing strategies and their potential impact on health. A youth participant expressed concern, saying “**I’m shocked and appalled. I think they’re setting us up”,** while another similarly expressed surprise, saying it **“slapped me in the face”**. A few participants stated that reading the booklet encouraged them to do further research to better understand targeted marketing. Overall, post-sensitization discussions focused on the new awareness that business strategies of the food industry can impact health, and that the Black community might be experiencing this effect in a more detrimental way. When asked by the Moderator, *“what would make you/others want to get involved in changing the way foods and beverages are marketed to us in our community?”,* participants often cited need for raising awareness of health impacts of certain foods and the use of targeted marketing to promote less healthy options, through educational activities. For instance, one adult participant said, **“I think maybe educating people on you know, how do you take the first steps in something like this”**. And another adult talked about reaching children: **“You’ve got to teach nutrition. It should be part of the education system to the extent that kids are taught to count calories just like they do green, yellow, and red”.** Others talked about specific approaches to impact the food environment, as mentioned by this adult participant: **“they (community members) can make a difference if they can find space in communities that’s willing to give them space to put their larger groceries in there”.** Other potential approaches to increase healthy eating were drawn from observing other communities, including gardening programs, information exchanges, and calorie labeling on menus.

#### 3.2.4. Direct Assessment of Responses to the Booklet and Relative Theme Prominence Pre- and Post-Sensitization 

Participants indicated, in response to direct questions, that the sensitization booklet increased their awareness of and interest in previously unrecognized issues associated with food marketing (Table 3).

**Figure 2:**
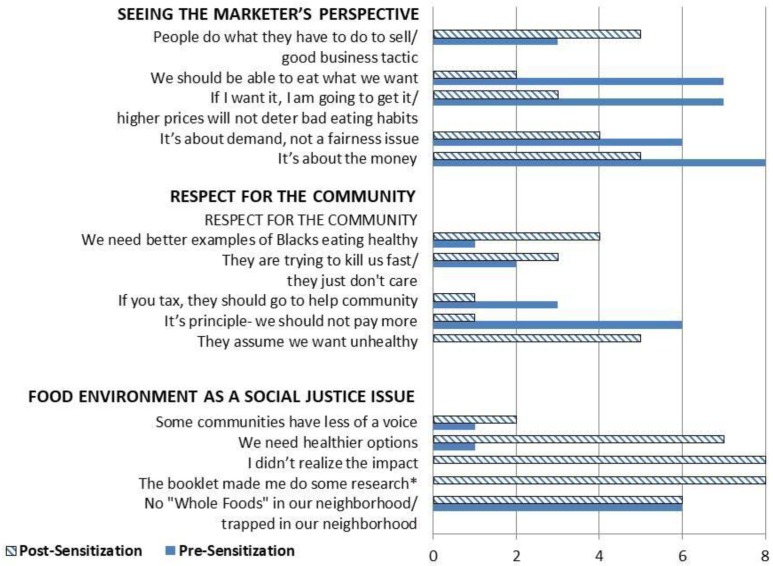
Number of focus groups in which selected codes representing the three main themes occurred before and after sensitization. See text for explanation.

As described in Methods, the initial impressions of changes in participant views and reactions also were evaluated by tabulating the pattern of code use pre- and post-sensitization. The initial step involved identifying codes that met two eligibility criteria: (a) they were used only for pre- or only for post- within a given focus group; and (b) they were used in more than one of the eight focus groups. The summary results of this analysis are shown in Figure 2, and provided in detail in an appended table (see Table S1). Examining the total occurrences of each code for each theme, leads to three main observations: (1) most of these selected codes were used both before and after sensitization; (2) the only codes that occurred solely after sensitization were associated with the ***Food environments as a Social Justice Issue*** theme, noting that one of these was directly related to a question about sensitization, and so could not have occurred in the pre-sensitization groups; and (c) with one exception (“people do what they have to do to sell/good business tactic”), the codes associated with ***Seeing the marketer’s perspective*** were applied more in the pre- vs. post-sensitization groups. A more mixed pattern of pre–post changes was seen for codes associated with ***Respect for Community***. This analysis also permitted assessment of where, i.e., in which groups, changes occurred, based on examination of the tabulations within each theme for each focus group as it applies to the codes in Figure 2 (see Table S1 for details). In summary, the code use for ***Seeing the marketer’s perspective*** was similar from pre- to post- in Durham; whereas it was used less after sensitization in all four of the Maryland groups. For ***Respect for Community*,** the code use was greater post-sensitization for male youth in both Durham and PG County and less post sensitization for adults in Maryland. For ***Food environments as a Social Justice Issue,*** use of all codes increased post-sensitization within all eight groups.

## 4. Discussion

When presented with depictions of targeted marketing approaches, including promotions, placement, pricing strategies, and tailored products, both Black youth and adults responded with varied lenses. In themes identified, some participants viewed marketing practices as being expected and understandable, because they aligned with good business practices that aim at getting the most profits possible. Profitable business strategies were not universally viewed as negative, as it was felt that profitable businesses could be good for the local community and/or offer access to products which might be desired by the community. Participants also endorsed the role of individual responsibility in determining how detrimental or influential marketing tactics were, where beliefs that consumers needed to choose better were asserted. Conversely, some themes evoked expressions of frustration and concern for the Black community at large, and particularly for children, older adults or those with additional constraints, such as lower income or being short on time (e.g., single parents). These concerns came through even more after the participants were given the booklet to sensitize them to common food and beverage marketing practices. They led to discussions of a need for more government oversight, more education on nutritional issues in the community, and more community advocacy to circumvent the marketing strategies they identified as leading to poor dietary quality and related negative health effects.

The themes identified, for the most part, were seen across both pre- and post-sensitization. Themes oriented to seeing social injustice in food and beverage marketing strategies were present before and after sensitization, although were more salient in post-sensitization. Conversely, themes sympathetic towards the need for companies to make money were more evident in pre-sensitization focus groups, although not absent from post-sensitization groups. Thus, the sensitization process appears to have increased negative views towards marketing approaches for some participants. These findings align with a small marketing study that assessed effects of participation in an educational workshop on media literacy on parental media literacy and perceptions/attitudes about unhealthy and healthy foods among children [22]. The author reported that media literacy did change: both children and parents became more critical of advertisements for unhealthy food.

Considering the objective of this study, the identified themes provide insight on potential approaches for mobilizing community action to increase demand for a healthier mix of products in the Black community. Participants’ comments supported efforts that would potentially tax less healthy foods if the revenue went to educate the community on nutrition. They also stated that the government could play a larger role in reducing the extent of some targeted marketing to which the community is exposed. Last, they described the need for community members to begin to advocate to reduce marketing approaches that increase access to less healthy options and to promote approaches which lead to greater access to healthy foods.

Lobstein and colleagues identified the roles of corporate and governmental entities in developing policies to reduce obesity, and described the role of advocates in identifying policies that support, rather than obstruct, healthy eating [23]. Advocates would work to become stakeholders in decision-making, regarding corporate practices and governmental policies which are impeding progress. We located one study that identified the need for sensitizing Black community advocates to their potential role in obesity prevention [24]. The study surveyed members of an advocacy organization whose mission is to advance quality of life for the Black children. Most respondents felt that parents were the primary responsible party in terms of obesity prevention, while far fewer felt food marketing was primarily responsible for poor eating in children. Authors of that study felt that advocates needed, in part, to be armed with knowledge regarding determinants of obesity. The current study suggests that combining efforts to increase media literacy with sensitization to food marketing tactics may be an effective way to encourage community advocacy for changes in marketing patterns.

This study on the issue of targeted marketing has several strengths, particularly, its relevance to Black Americans, a population group at high-risk for obesity and related chronic diseases. Black American ethnicity has been, and continues to be, a major focal point for targeting of unhealthy products to adults, youth, or both—not only high-calorie, nutrient-poor foods and beverages, but also alcohol and tobacco [8,25,26]. Our findings add to the broader literature on targeted marketing, how consumers respond to it, and the particular salience of marketing to groups who can identify themselves as being targeted based on ethnic or other observable group-specific characteristics [11]. Although unhealthy products are marketed to many audiences, efforts to mitigate the harmful effects of ethnically targeted marketing must be population specific. The sensitization study design may be adaptable for other populations that are targeted because of their recognizable characteristics. The participants were intentionally recruited in partnership with organizations in their communities to increase the trust in information presented. The use of an anonymous polling procedure in the initial focus groups was thought to increase the potential for honest responses on which to build during discussion.

With respect to the analytic approach, the conventional content analysis performed by two researchers offered a reliable and valid method of identifying emergent themes from the data. The coding approach allowed for codes to occur across pre- and post-sensitization groups, so that analysis would lead to an assessment of how sensitization influenced the emergent themes. The computer assisted coding process facilitated various ways of examining the findings, including the tabulation of codes which confirmed the initial impressions of changes in salience of themes, pre- and post-sensitization. Last, the sensitization process was novel for this food marketing topic.

The qualitative approach allowed for depth of findings on this topic for which there is limited empirical data in the public health literature. However, the findings should be considered in light of limitations of this study, which include the predominantly female sample among the adults (limiting the ability to understand perspectives of males), the potential for selection bias favoring an interest in food marketing issues, and possible lower applicability of relying on a booklet approach to people with limited literacy. With respect to selection bias, this does not necessarily detract from the utility of these results, given that people interested in the topic would be those most likely to become engaged in related advocacy efforts. The samples were small, although engaging with both adult and youth participants in two different geographic areas provided for diverse views. The method of sensitization and a post-sensitization focus group allowed for participants to have more time to think about questions posed and discuss with others between sessions, although there is no way to determine the occurrence or influence of such interactions outside of the focus group setting. The paper booklet offered a good method to engage with adults and youth of varying economic/age levels who might have varying access to technology (e.g., young teens might not have had their own mobile phone) but would be less effective in the context of lower literacy. Access to mobile phones with “smart” applications has opened the door to utilizing technology for both polling and to deliver a sensitization, which increases feasibility, potentially allows for more dynamic and tailored presentations of sensitization data, and facilitates tracking of participant engagement/interaction with the sensitization material.

## 5. Conclusions

In light of these issues, the current findings can be used to encourage the use of sensitization procedures to heighten awareness of marketing tactics which may negatively influence food choices of Black adults and youth. Such approaches could be introduced in research interventions and into policy approaches. Others have suggested that policy approaches to reducing obesity should be tailored to the community in terms of food preferences, social factors, and economic constraints [27]. Thus, policies developed to address race/ethnicity-related health disparities should incorporate opportunities to address individual constraints around purchasing a healthier mix of foods, while also looking at ways to create an environment that promotes and facilitates these purchases.

## Figures and Tables

**Table 1:** Scenario descriptions and associated questions from moderator guide for pre-sensitization focus groups with adults and youth.

Adult Focus Groups	Youth Focus Groups
Scenario Described (Promotion): This past summer, (Fast Food Company) sponsored a national tour, visiting six U.S. cities. Headlined by a (Popular R and B Singer/TV Actor Name). Question Posed: *Do you see this as marketing? (Clicker answer options: yes/no/unsure)*	Scenario Described (Promotion): (Popular Rap Artist Name) will perform for Black teens in an exclusive, free concert sponsored by (Large Beverage Company) Question Posed: *Do you see this as marketing? (Clicker answer options: yes/no/unsure)*
Scenario Described (Place): Supermarkets are not always located in every neighborhood. Questions Posed: *Do you know of neighborhoods that don’t have supermarkets? (Clicker answer options: yes/no/unsure)*; *Smaller neighborhood stores tend to have higher prices than large supermarkets or “big box” stores. Do you think this is fair to the consumer? (Clicker answer options: yes/no/unsure)*	Same as for adult groups
Scenario Described (Place): Last year the Los angeles city council passed a new law to prohibit the opening of new fast food restaurants for one year in South Los Angeles neighborhoods. *Questions Posed: Do you think it would be good to pass a law like this that would affect your neighborhood? (Clicker answer options: yes/no/unsure)*; *Do you think it should be up to public officials to determine what types of restaurants or food stores are available in your neighborhood? (Clicker answer options: yes/no/unsure)*	Scenario Described (Place): A survey of food prices found that the stores near the mostly Black high schools had higher prices for items like potato chips than stores near white schools. Questions Posed: *If you found this to be true in your area, would this matter to you? (Clicker answer options: yes/no/unsure)’; Now a community group is trying to stop the stores from selling so many of these snack foods and to stock more, healthier products. If you found this to be true near your school, would it matter to you? (Clicker answer options: yes/no/unsure)*
Scenario Described (Price): Several states have a specific sales tax on soft drinks and other sugar-sweetened beverages. Such taxes apply to sports drinks, sweetened teas, fruit drinks, but do not apply to diet sodas or 100% fruit juice. Question Posed: *Where would you stand on this issue (soda tax)? (Clicker Answer options: In favor of the tax; In favor of the tax under some circumstances, i.e. how the revenues from the tax would be utilized; Not in favor of the tax; unsure*	Same as for adult groups
Scenario Described (Productand Promotion): A new research report summarizes Black Americans’ food-related attitudes and behaviors. The report states that Black Americans have less interest in healthy eating relative to the population as a whole, and that companies can profit by targeting Black households for products like chips and soda, which other households are eating less of. Question Posed: *Does it matter to you that companies target you to sell certain foods and beverages? (Clicker answer options: yes/no/unsure)*	Same as for adult groups
Scenario Described (Product): Research shows that Black consumers often add sugar to their tea when it is already sweetened. A fast food company decides to offer a sweeter version of the tea at locations in Black areas and promote regular tea in other areas. Question Posed: *Do you think people should be told that they are getting a different product? (Clicker answer options: yes/no/unsure)*	Same as for adult groups
Scenario Described (Product): Products can be designed to appeal to different types of consumers, for example cigarette brands, flavors, and packages have been designed to appeal to youth. Question Posed: *Which of the following best describes your opinion about this? (Clicker answer options: “This won’t affect children if they know better than to smoke.”; “I think this is a normal marketing tactic.”; “I don’t think companies should try to sell)*	Scenario Described (Product): Products can be designed to appeal to different types of consumers, for example cigarette brands, flavors, and packages have been designed to appeal to youth (people your age). Question Posed: *Which of the following best describes Your opinion about this? (Clicker answer options: “This won’t affect children if they know better than to smoke.”; “I think this is a normal marketing tactic.”; “I don’t think companies should try to sell cigarettes to children”; “None of the above describes my opinion”)*
Scenario Described (Promotion):(Large Beverage Company) unveiled the (Phone App Name) for mobile phones, to create a “community-to-go” and to interact with its “mostly African-American youth target audience”. Question Posed: *Do you think this type of program helps companies sell their products? (Clicker answer options: yes/no/unsure)*	Same as for adult groups
Scenario Described (Place/Promotion): The number of outdoor ads has been found to greater in primarily African American areas Question Posed: *Do you think that the amount of outdoor advertisements in African American neighborhoods influences what people buy? (Clicker answer options: yes/no/unsure)*	Same as for adult groups
Scenario Described (Price/Place): A survey of food prices found that the stores near the mostly Black high schools had higher prices for items like potato chips than stores near white schools. Questions Posed: *If you found this to be true in your area, would this matter to you? (Clicker answer options: yes/no/unsure)’; Now a community group is trying to stop the stores from selling so many of these snack foods and to stock more, healthier products. If you found this to be true near your school, would it matter to you? (Clicker answer options: yes/no/unsure)*	(Presented earlier in youth group)
Scenario Described (Promotion): TV and radio commercials are designed to appeal to different types of consumers. For example, commercials are designed to appeal to different racial or ethnic groups. Question Posed: *Does it matter to you if advertisers use your ethnicity or cultural ties to try to sell you products and services? (Clicker answer options: yes/no/unsure)*	Same as for adult groups
Scenario Described (Promotion): Marketing agencies often use popular artists’ songs to promote specific product brands for cars, clothes, beverages, etc. Question Posed: *Do you think this type of ad influences what people buy? (clicker answer options: yes/no/unsure) In relation to food and beverages, what types of products would you like to see advertised in your community? (open ended responses only)*	Same as for adult groups

Note: Scenarios were developed with the goal of highlighting examples of promotion, pricing, placement, and product development that are designed to influence purchases. They are organized in this table based on the sequence in which they were presented to the participants.

**Table 2:** Demographic data for adult study participants (n = 30).

Variable	Durham Participants	Prince George’s County Participants
	n = 10 *	n = 15
Age		
19 to 35 years	7 (70%)	3 (20%)
36 to 65 years	3 (30%)	12 (80%)
Female Male	8 (80%) 2 (20%)	12 (80%) 3 (20%)
Education		
Some college or less	8 (80%)	7 (47%)
College grad or more	2 (20%)	8 (53%)
Married/living with partner	1 (10%)	8 (53%)
Employment		
Full or part-time	5 (50%)	11 (79) **
Not employed, retired/student	5 (50%)	3 (21%)
Primary household shopper	6 (60%)	11 (73%)

*: 5 of the 15 Durham participants did not complete the questionnaire; thus, percentages shown for Duke are based on the 10 participants who completed these forms. **: Based on n = 14; 1 participant did not answer the employment question.

**Table 3:** Examples of participant comments at the follow up focus group in response to moderator probes about their reactions to the information in the booklet.

Site, Gender, and Age Group	Comments
Durham, Adults	(General Reaction) “And as I read the book… Then later on I was looking in the different communities… and seeing that a lot of the advertisement, the posting, the pricing, you know, everything that was in the book was just like it was in real life.”
Durham, Boys	(General Reaction) “I just feel like it’s surprising out here you try to, you know, companies try to get in where they fit in, I guess. From a company point of view I could say it’s a very smart way to market, you know, to find out what people like and then try to persuade them through the things that they like, but just on the other hand I just feel like there’s something about it that’s shady.”
Durham, Girls	(In reaction to Promotion section) “like every time (TV CHANNEL aimed at Black audience) a commercial it’s for (FAST FOOD RESTAURANT). So that makes—that makes sense. But the way that they do it you really don’t notice it. You know? Because you’re just watching TV. You’re like oh, that looks good… So it’s—it’s really slick.… That’s interesting.”
Prince George’s County, Adults	(In reaction to Price section) “What stuck out to me was the value meals, you know, supersizing and things like that. And actually it made me, the source, I went on the internet and looked it up, and it was like a 14-page report on it. And it was just amazing to me how they, they market”.
Prince George’s County, Boys	(In reaction to Place section) “I mean not just in the book but I didn’t really know that not everybody had a supermarket. I thought everyone did. I didn’t—that was like I don’t know.”
Prince George’s County, Girls	(In reaction to Place section) “the smaller-the smallest store is carrying, um, less fresh produce. I never really thought about that, but it actually makes more sense now.”

## Supplementary Materials

The following are available online at www.mdpi.com/1660-4601/14/11/1316/s1, Table S1: Comparison of use of selected codes within the three themes pre-and post-sensitization by focus group within and across study sites.
