# Supplementary material for: Sensitizing Black Adult and Youth Consumers to Targeted Food Marketing Tactics in Their Environments
Source: Int J Environ Res Public Health. 2017 Oct 29;14(11):1316. doi: 10.3390/ijerph14111316 (PMC5707955; doi:10.3390/ijerph14111316)
Supplement: Supplementary file 1 [file ijerph-14-01316-s001.pdf]

African American Collaborative Obesity Research Network

This booklet was developed by the African American Collaborative Obesity Research Network and collaborators at the University of Maryland (Dr. Deborah Rohm Young & staff) and at Duke University (Dr. Lori Carter-Edwards & staff).

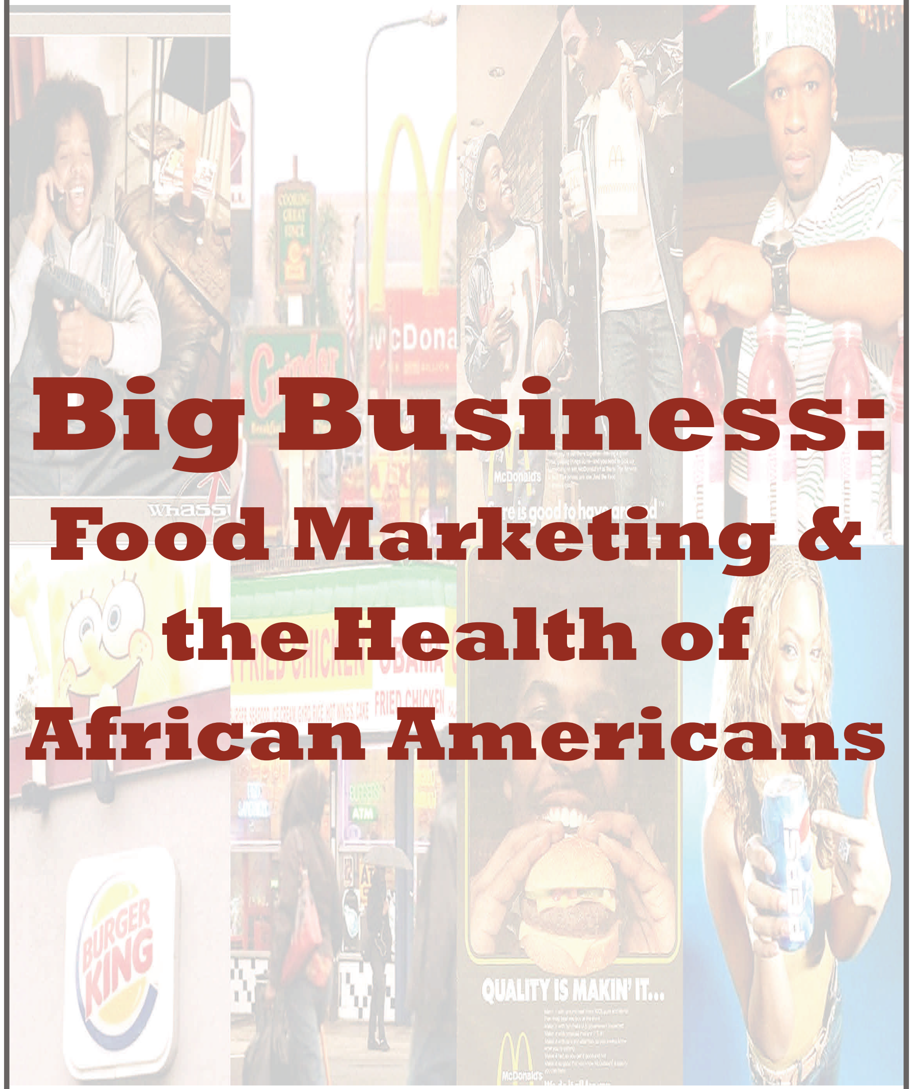

Big Business:

Food Marketing & the Health of African Americans

There are two sides to every story. This is true when we decide if the way products are marketed to us is "fair" or possibly harmful. We chose the examples during the focus group because they represent marketing strategies. Scientific studies and other reliable sources suggest some marketing is "unfair" or possibly harmful. We are asking you to think more about marketing between now and the next group session.

SUPERMARKETS

* Several studies show that far fewer **African American neighborhoods have a supermarket than mostly white neighborhoods.**
* If supermarkets are not close to where people live, people might have to shop at small neighborhood stores. They will get less quality food for their money. They also will have fewer fresh food choices. A study found the average Philadelphia teenage student purchases more than 350 calories on a daily trip to the neighborhood store. This means that **in 1 month, a teen might buy 10,000 calories worth of food from their neighborhood store.**

SOURCES: 1.. *Food store availability and neighborhood characteristics in the United States*. Published in Preventive Medicine (2007). Authors: Powell, Slater, Mirtcheva, Bao, & Chaloupka. ***3.*** *Snacking in Children: The Role of Urban Corner Stores*. Published in Pediatrics (2009), Authors: Borradaile and Colleagues.

What happens if Supermarkets are opened in neighborhoods that don't have them?

* Fruit and vegetable consumption increases by 32% for African Americans with each new supermarket in their neighborhood.
* Supermarkets also bring jobs and stability to neighborhoods.

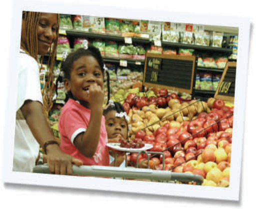

SOURCE: *The Grocery Gap: Who has access to healthy food and why it matters***,** Authors: PolicyLink and The Food Trust.

Place

What it is: Place is the location where a product can be purchased.

How Food Marketers Use it

The location of food stores and restaurants can affect food's price, freshness, and availability.

Examples of Place:

FAST FOOD RESTAURANTS

* In a study of fast food restaurants in white versus black neighborhoods, **mostly black neighborhoods have over twice the number of fast food restaurants.** Most fast food is high in fat and calories. Eating often at fast food restaurants can result in weight gain and obesity.

SOURCE: *Fast food, race/ethnicity, and income: A geographic analysis.* Published in the American Journal of Preventive Medicine (2004), Authors: Block, Scribner, & DeSalvo.

SMALL STORES

* **Small stores tend to carry less fresh foods.** Processed foods like chips and soda can stay on the shelf for a long time. Fresh foods might spoil before they sell. This makes fresh foods less money-making for the small stores.

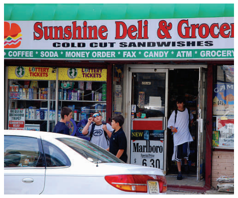

There are four sections to this booklet. They cover the "Four P's" of marketing: Product, Price, Promotion, and Placement. The "Four P's" are the tools that marketers use to connect their products and services to people who purchase them. We are sharing information on how food companies market their products to consumers using the "Four P's."

We want to get your opinions on these examples of food marketing practices. They may be linked to high calorie consumption, which can lead to obesity. They can also lead to higher rates of illnesses, like heart disease and diabetes.

We look forward to hearing your thoughts at the next group meeting!

Product

What it is: Product is an item offered to the customer. How a product is packaged, how it looks, and how it's used are all considered when creating and marketing food products.

How Food Marketers Use it:

* **Product taste, packaging, and appearance** are important product factors. Taste is one of the major reasons people buy food. Others might choose a product because of the package or the appearance of the product. For example, cereal boxes often show kid-friendly images.
* **Smell ("aroma")** is also important. Recognizing a product by its aroma might drive some people to buy it. For example, the smell of baked goods can be appealing to shoppers.
* **Reliability and convenience** also get consumers to buy a product. Some people purchase packaged food products because they are reliable and save us time. A particular brand of packaged chocolate chip cookies will always tastes the same. Also, these cookies might be more convenient because they save us time than if we were to bake ourselves

Examples of Promotion:

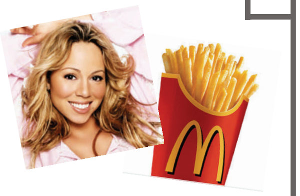

Promoting Products through Placement in Songs

* If you have heard Mariah Carey's song, "More than Just Friends," she sings about a friend that she likes. The song goes *"ya like a little model; I'm steady feenin' for ya like them fries at McDonald's"*.
* McDonald's pays Hip-Hop artists $1-5 for every time a song plays on the radio that references McDonald's. It is not known which artists they are paying. But we know that songs with food products in the lyrics are a new placement strategy.

SOURCES: 1. *McDonald's Buying Way into Hip-Hop Song Lyrics*, Published in Advertising Age (2005). Author: Graser, M. **2.** *Hip-hop Gets Down with the Deals*, Published in Business Week Online (2005). Author: Kiley, D.

Promoting Food Products through Targeting African American Programming

A study of TV commercials showed that black prime time shows had almost twice as many food commercials compared with general audience programs. There were **40% more fast food commercials on the top 10 African American TV programs** compared to general audience programs.

SOURCE: *Food Advertising in the Age of Obesity: Content Analysis of Food Advertising on General Market and African American Television.* Published in the Journal of Nutrition Behavior and Education (2005), Authors: Henderson & Kelly

Promotion

What it is: Promotion is the information communicated to possible consumers. It includes advertising, public relations (company public image), sales promotions, and sponsorship of events (like music concerts).

How Food Marketers Use It:

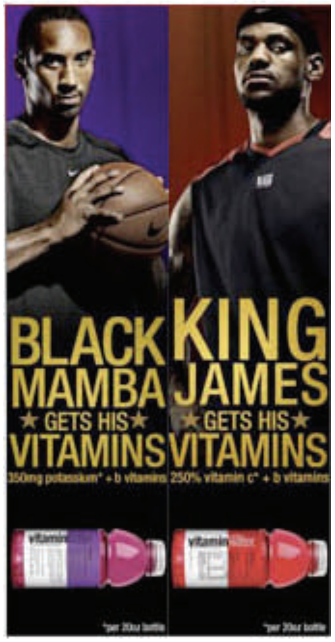

* Advertisements are used to make the consumer familiar with the product. They are also used to encourage brand loyalty. Ads are found in magazines and newspapers, radio and television commercials and promotional events (such as sponsored music concerts).
* Marketers use celebrities, such as movie stars, musicians, and athletes, to promote products. Showing a celebrity using a product might make a product more popular with fans. This can help sell the product.

Targeting of Certain Food Products to African Americans

Food and Beverage products are developed to target one or more market segments, such as African Americans and Hispanics. In developing targeted products for African Americans, food or beverage companies try to make products appeal to African American tastes, such as specific flavors, packaging, or portion sizes. The beverage industry has particularly focused on African Americans as preferring sweeter, fruit-flavored beverages with less carbonation.

SOURCE: *Sweet Spot; Soda marketers answer demo shift by aiming to woo Hispanic and Black teens*. Published in Advertising Age (2001). Author: Chura, H.

An Example of Products Targeted for African American Communities

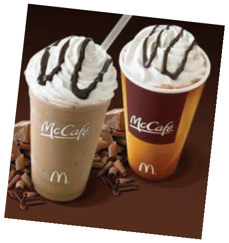

Sweeter Drinks

Market Researchers found that African Americans like their drinks, such as teas and coffees, sweeter than other population groups. So the food companies made sweeter products and sold them in African American areas. An example of this is McDonald's McCafé® drinks, like the Real Fruit Smoothies and the Iced Mochas. The Chief Marketing Officer of McDonald's (USA) said in an interview that these sweeter tasting drinks appeal to blacks.

SOURCE: Ethnic Marketing: McDonald's Is Lovin' It. Published in Business Week (2010). Author: Helm, B.

Price

What it is: Price is the amount a customer pays for a product. Price includes the dollar cost, time, and effort a consumer might spend to buy a product. Price is decided by what the customer thinks the product is worth. It is also based on other nearby store prices and the product's demand.

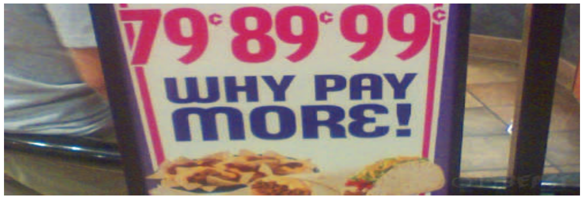

How Food Marketers Use it:

* A business may decrease the price of a product if most other stores also carry it. If other stores do not carry it, they might raise the price.
* For restaurants, price is used to bring in the customer. Advertising often focuses on low prices and good value. Combo menus were created for fast food restaurants to encourage a larger purchase. Customers leave the restaurant believing they are getting a "**good deal**".
* The government uses price in the form of taxes to discourage purchasing of harmful products. Taxes are used with Cigarettes, Tobacco, and Alcohol.

Examples of Price Affecting Purchasing:

Value Meals: Fast Food Restaurants offer size upgrades and "value meals" to sell more food at a bargain price. They can do this because the actual food does not cost them alot. The costs of labor, packaging, and marketing are greater business expenses. A consumer might be focused on the food value and not think of the additional calories that come with that value. For example, choosing a "value meal" that includes a large-sized soda and french fries can add 400 calories to a fast food meal.

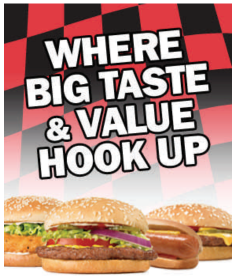

SOURCE: *The National Alliance for Nutrition and Activity (NANA) report titled:* " From Wallet to Waistline: The Hidden Costs of Super Sizing."

Soda Tax: Some states now tax soda and other sweetened drinks to help combat obesity. Such taxes are put on non-diet sodas, sports drinks, fruit drinks, sweetened teas, and Kool-Aid. Diet sodas or 100% fruit juices are NOT taxed. The idea is that taxing sugary drinks will raise the price and people may choose to not buy them. Or, since many people might still buy sodas, the tax could be a way to help close government budget gaps.

SOURCE: *Soft drink tax battle shifts to states.* Published in the Los Angeles Times (2010). Author: Geiger, K. & Hamburger, T.

NOTES & REACTIONS

Table S1 Comparison of use of selected codes within the three themes pre-and post-sensitization by focus group within and across study sites

Selected codes within each theme[1]	North Carolina								Maryland								Pre- and Post-totals across the 8 Groups
	Adults (1)		Adults (2)		Female Youth		Male Youth		Adults (1)		Adults(2)		Female Youth		Male Youth		
	Pre	Post	Pre	Post	Pre	Post	Pre	Post	Pre	Post	Pre	Post	Pre	Post	Pre	Post	
**Seeing the marketer's perspective** ("it's about demand"; "consumers choose")																	
It's about the money	×	×	×	×	×	×	×	×	×	×	×		×		×		Pre: 8<br>Post: 5
It's about demand, not a fairness issue	×	×	×	×	×	×		×	×		×		×				Pre: 6<br>Post: 4
If I want it, I am going to get it/Higher prices will not deter bad eating habits	×			×	×	×	×	×	×		×		×		×		Pre: 7<br>Post: 3
We should be able to eat what we want	×	×	×		×		×		×		×		×	×			Pre: 7<br>Post: 2
People do what they have to do to sell/Good business tactic		×				×	×	×					×	×	×	×	Pre: 3<br>Post: 5
**Column Totals**	4	4	3	3	4	4	4	4	4	1	4	0	5	2	3	1	
**Respect for the community** ("marketers are setting us up for failure"; "making wrong assumptions")																	
They assume we want unhealthy		×				×		×		×				×			Pre: 0<br>Post: 5
It's principle- we should not pay more					×		×		×		×		×		×	×	Pre: 6<br>Post: 1
If you tax, they should go to help community									×		×		×			×	Pre: 3<br>Post: 1

They are trying to kill us fast/ They just don't care	×				×			×						×		×	Pre: 2<br>Post: 3
We need better examples of Blacks eating healthy	×	×				×				×		×					Pre: 1<br>Post: 4
Column Totals	2	2	0	0	2	2	1	2	2	2	2	1	2	2	1	3	
**Food environments as a social justice issue (**"no one is watching the door"; "I didn't realize")																	
No Whole Foods in our neighborhood/trapped in our environment	×	×			×	×		×	×	×	×	×	×	×	×		Pre: 6<br>Post: 6
The booklet made me do some research[2]		×		×		×		×		×		×		×		×	Pre: 0<br>Post: 8
I didn't realize the impact		×		×		×		×		×		×		×		×	Pre: 0<br>Post: 8
We need healthier options		×		×				×	×	×		×		×		×	Pre: 1<br>Post: 7
Some communities have less of a voice										×	×			×			Pre: 1<br>Post: 2
**Column Totals**	1	5	0	3	1	3	0	4	2	5	2	4	1	5	0	3	

[1] Codes were selected for this analysis if the a) had been used in more than one focus group and if they had be used only pre- or post-sensitization in at least one focus group. Thus, codes that, when used, had always been used both pre- and post-sensitization in the focus groups for which they were used, are not included in this worksheet.

[2]Note: This code was directly related to response to the sensitization booklet, so it could not occur in the pre-sensitization focus group.

Changes from pre- to post-sensitization are highlighted in green.
